# Occurrence, Morphometric, and Molecular Investigation of Cattle and Buffalo Liver Adult Fluke in Peninsular Malaysia Main Abattoirs

**DOI:** 10.1155/2020/5436846

**Published:** 2020-06-12

**Authors:** J. Nur Alia Diyana, M. I. Nur Mahiza, H. Latiffah, S. H. Nur Fazila, I. H. Lokman, H. Noor Hazfalinda, P. Chandrawathani, E. B. Ibitoye, K. Juriah

**Affiliations:** ^1^Faculty of Veterinary Medicine, Universiti Putra Malaysia, 43400 Serdang, Selangor, Malaysia; ^2^Faculty of Health Sciences, Universiti Kebangsaan Malaysia, 43600, UKM, Bangi, Selangor, Malaysia; ^3^Research and Innovation, Department of Veterinary Services, 62624 Putrajaya, Malaysia; ^4^Department of Theriogenology and Animal Production, Faculty of Veterinary Medicine, Usmanu Danfodiyo University, Sokoto, Nigeria; ^5^Faculty of Agriculture and Food, Universiti Putra Malaysia Bintulu Sarawak, 97008 Sarawak, Malaysia

## Abstract

Fascioliasis is a parasitic disease of human and animal caused by *Fasciola gigantica* (*F. gigantica*) and *Fasciola hepatica* (*F. hepatica*). More than 700 million of grazing animals and over 180 million human population are at the risk of fascioliasis. This study was conducted in Banting, Ipoh, Shah Alam, and Taiping abattoirs within Peninsular Malaysia to determine the occurrence and identify the species of liver flukes, causing liver condemnation in cattle and buffaloes. Within a study period from January to December 2018, a total of 25 condemned bovine livers were collected from Banting, Ipoh, Shah Alam, and Taiping abattoirs and analyzed. Taiping abattoir had the highest occurrence of fascioliasis [14/1014 (1.38%)], and Shah Alam abattoir had the least occurrence [1/3377 (0.03%)]. From all the sampled livers, the average number of adult flukes recorded ranged from 1 to 83. A total of 440 adult flukes were studied morphometrically, using parameters such as body length (BL), body width (BW), cone length (CL), cone width (CL), body area (BA), body perimeter (BP), the distance between the ventral sucker and posterior end of body (VS-P), BL/BW ratio, BL/V-SP, and body roundness (BR). Furthermore, molecular analysis was conducted using PCR-RFLP methods to distinguish between *F. gigantica* and *F. hepatica* involving ITS1 primer and RsaI restriction enzyme. RFLP pattern with RsaI produced a consistent pattern of 360,100 and 60 bp fragments in *F. hepatica*, whereas *F. gigantica* worms had a profile of 360,170, and 60 bp in size. The morphometric and molecular analysis results indicated that cattle and buffaloes slaughtered at Banting, Shah Alam, Taiping, and Ipoh abattoirs were infected with *F. gigantica*.

## 1. Introduction

Fascioliasis is common among grazing animals in most parts of Malaysia, and its prevalence can reach up to 50% in some provinces [1]. *F. gigantica* and *F. hepatica* are the two aetiologic species of fascioliasis [2]. *F. hepatica* is widely distributed worldwide, but *F. gigantica* is distributed primarily in the tropical and subtropical regions [3]. It is essential to differentiate between species of *Fasciola*. Although intermediate host for both species (*F. hepatica* and *F. gigantica*) vary in terms of biological and ecological characteristics, intermediate forms between the two *Fasciola* species have been extensively reported in shared hosts in Korea [4], Japan [5], Vietnam [6], China [7], and Iran [8] [9]. In some countries in Asia, *F. hepatica* and *F. gigantica* coexist, and the intermediate fasciolid forms of adults usually exist in the same host. Due to coexistence, the similarity in intermediate fasciolid forms, and difficulties in differentiating the species, these flukes of either species are generally classified as *Fasciola* sp.

Morphological characteristics such as the ratio of body length to width can be used to differentiate these two species. However, it is difficult to be done due to variations in their sizes, involving the age of the flukes, host species, and fixation techniques used [10]. The morphological method has a limitation on the differentiation of *Fasciola* species as the value may be overlap. Thus, several molecular analysis using different molecular targets have been developed for the differentiation of *F. hepatica* and *F. gigantica*. DNA sequencing of first internal transcribed spacers (ITS1), ITS2, and 28S ribosomal ribonucleic was recognized as a molecular approach to differentiate *Fasciola* species [11].

In Malaysia, there is a lack of information on the morphometric and molecular characterization of adult *Fasciola* forms, and this has limited the exact identification of adult fluke species obtained from slaughterhouses. This study, therefore, evaluated the occurrence of fascioliasis from Banting, Ipoh, Shah Alam, and Taiping abattoirs in Malaysia and identified adult *Fasciola* species obtained from the liver sample collected from these abattoirs via morphometric and molecular analysis.

## 2. Materials and Methods

### 2.1. Collection of Adult Flukes

**Figure 1:**
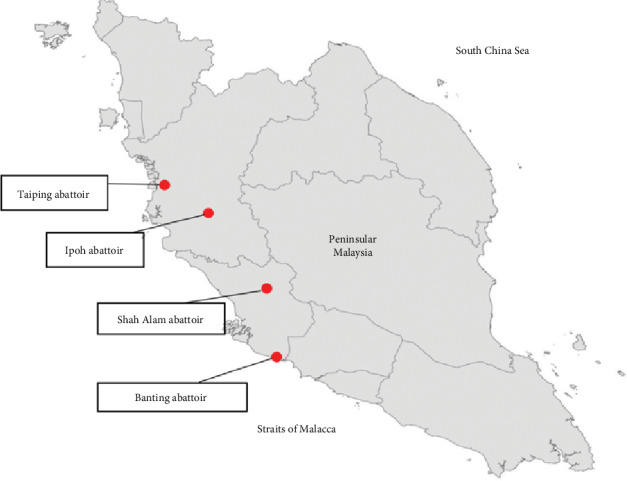
The location of abattoirs where all the condemn liver collected.

Using convenience sampling, condemned livers due to fascioliasis were collected from Banting, Ipoh, Shah Alam, and Taiping abattoirs from January to December 2018 (Figure 1). The livers were transported back to Parasitology laboratory, Faculty of Veterinary Medicine, Universiti Putra Malaysia (UPM), using icebox immediately after sampling. Adult flukes were extracted from the livers using forceps and were transferred into petri dishes containing 0.9% saline solution. Next, the adult flukes were soaked in petri dishes containing soda water to relax their body and prevent them from curling up or forming wrinkles on the surface. A few hours later, the adult flukes were preserved in glass jars containing 75% ethanol prior to morphometric examination.

### 2.2. Morphometry Study

All standardized measurements of adult flukes were made according to methods previously proposed [12]. Morphometric values for body length (BL), body width (BW), cone length (CL), cone width (CL), body area (BA), body perimeter (BP), the distance between ventral sucker and posterior end of body (VS-P), BL/BW ratio, BL/V-SP, and body roundness (BR) were measured using a calliper. Body roundness (BR = BP^2^/4*π*BA) was used to compute the body shape by measuring how circular the fluke is. A circular object will have a roundness of 1.0, while irregular objects have larger values [13]. The results obtained from this study were analyzed through independent *T*-test and ANOVA using SPSS software. *T*-test was used to compare the mean of different variables between *F. hepatica* and *F. gigantica*, while one-way ANOVA was used to analyze differences between the means of morphometric values in flukes isolated from different hosts [1].

### 2.3. DNA Extraction

For the DNA extraction, a portion of the apical and lateral zone of adult flukes, not including the reproductive organs, were removed and crushed. Total DNA was extracted using QIAamp DNA Mini Kit (No. 51304, Qiagen, Germany) according to the manufacturer's instructions. Extracted DNA was diluted in double-distilled water and maintained at -20°C until it was used in the PCR.

### 2.4. PCR Amplification

For PCR amplification, at approximately 680 bp region of the ITS1 sequence, PCR was performed using a set of ITS1-F (5′-TTG CGC TGA TTA CGT CCC TG-3) [5]. A total volume of PCR reaction was 25 *μ*l, containing 7*μ*l of the DNA solution, 18*μ*l master mix (Thermo Fisher Scientific, USA), 1.0*μ*l of each primer, and 5.4 *μ*l of distilled water. The reaction cycle was as follows: one cycle of 90 s at 94°C, 30 cycles of 90 s at 94°C, 90 s at 55°C, 120 s at 72°C, and a final extension of 72°C for 10 min followed by cooling at 4°C.

### 2.5. Restriction Fragment Length Polymorphism (RFLP) Analysis

After incubation at 37°C for 3 hours and heat inactivation of RsaI at 65°C for 15 minutes, the digested DNA samples were analyzed by gel electrophoresis, 8*μ*l of each product, and were electrophoresed on 1.5% agarose gel in TBE buffer at 100 V for 60 minutes and visualized by UV illumination. The size of each band was determined by a 100 bp plus ladder molecular weight marker (Thermo Fisher Scientific, USA). DNA types of *Fasciola* spp. were distinguished according to fragment patterns, three bands of 360, 100, and 60 bp fragments in *F. hepatica*, while *F. gigantica* had a profile of 360, 170, and 60 bp in size [11].

## 3. Results and Discussion

Table 1 shows the occurrence of fascioliasis from the condemned livers collected from Banting, Ipoh, Shah Alam, and Taiping abattoirs. The highest occurrence of fascioliasis was recorded from the Taiping abattoir (14/1014), while the lowest occurrence was recorded from the Shah Alam abattoir (1/3377). It is likely that the lowest occurrence of fascioliasis in Shah Alam is due to Shah Alam abattoirs having larger throughput far above Taiping abattoir. In contrast, the high occurrence in Taiping may relate to its location in Perak, which is known to have the highest rainfall in Peninsular Malaysia. Taiping is recognized as the wettest area in Peninsular Malaysia with an average annual rainfall of ~4,000 mm, while the average rainfall in Peninsula ranges between 2,000 and 2,500 mm [14]. This higher rainfall could have favoured the proliferation of the intermediate host (*Lymnaea* sp.) [15] and triggered an increase in cases of fascioliasis around Taiping.

A total of 440 fasciolid worms were obtained from the infected livers from Shah Alam, Banting, Taiping, and Ipoh abattoirs and were analyzed. Ten different parameters pertaining to both *Fasciola* species were involved in the morphometric assessment of adult flukes in this study. Mean and range of the morphometric values of the 440 isolated adult flukes from cattle and buffalo are shown in Table 2. On average, eighteen liver flukes were collected from each liver out of 25 total condemned livers obtained in this study. From the morphometry study conducted, the mean value for all the parameters measured from adult fluke isolated from cattle was higher than those isolated from buffaloes (Table 2).

The results of the ANOVA showed that among the factors measured, except for BR, significant (*p* < 0.05) differences were present among BL, BW, CL, CW, BA, BP, VS-P, BL/BW ratio, and BL/V-SP (Table 2), across hosts. *T*-test also showed a significant (*p* < 0.05) difference in the size of BL, BW, CW, BA, BP, VS-P, BL/BW ratio, and BL/V-SP between flukes isolated from cattle and buffaloes. Based on the morphological criteria, all fasciolids isolated from this study were identified as *F. gigantica*.

The morphometric data of *F. gigantica* and *F. hepatica* from Egypt is shown in Table 3, as reported by Periago et al. [12]. Based on the result, the measured range values overlapped between cattle and buffalo although the mean of morphometric values in flukes from cattle were higher than those of buffaloes, and it was consistent with the previous report [12, 13] that stated that *Fasciola* in cattle and buffalo are morphologically identical.

The parameters for body length, body width, cone length, body area, body perimeter, VS-P, BL/BW ratio, BL/V-SP, and body roundness in this study are all suggestive of *F. gigantica* (Table 3). However, the cone width (CW) measurement was similar to *F. hepatica*. This situation might be due to the variabilities in the intensity of infection, host species, live parasite stage, and immune reactions from possible previous exposure to the disease [16]. In many cases, the phenotypic variations present in populations of free-living species become evident when they come from a different geographical location, or a pronounced change in their environment has taken place [12].

PCR-RFLP assay is a powerful method to distinguish between *F. hepatica* and *F. gigantica*. In this study, PCR-RFLP based on the partial rDNA of ITS1 and restriction RsaI enzyme were used to differentiate and identify the *Fasciola* species in Ipoh, Taiping, Banting, and Shah Alam abattoirs. This technique differentiates *Fasciola* species based on the profiles generated by the effects of endonucleases on the ITS genes of these parasites [17]. For example, the RFLP method was used in a few studies in Iran using DraI and BfrI for the 18s DNA region, TasI for ITS1 region, AvaII, and DraII for 28s DNA, and BamH1 and PagI for ITS2 [1].

**Figure 2:**
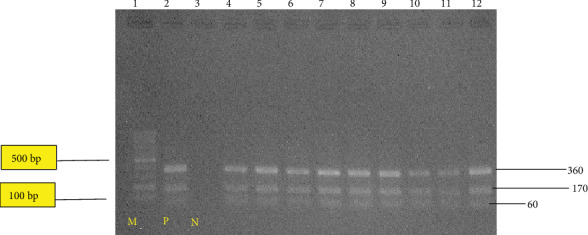
RFLP pattern of PCR product of liver fluke collected from bovine in Shah Alam, Banting, Taiping, and Ipoh abattoirs after digestion with RsaI enzyme. Lane 1: 100 bp ladder molecular; lane 2: positive control; lane 3: the negative control; lanes 4 to 20: denoted to those of *F. gigantica*. The PCR product digested were run on 2% agarose gel at 100 v for 60 minutes.

**Figure 3:**
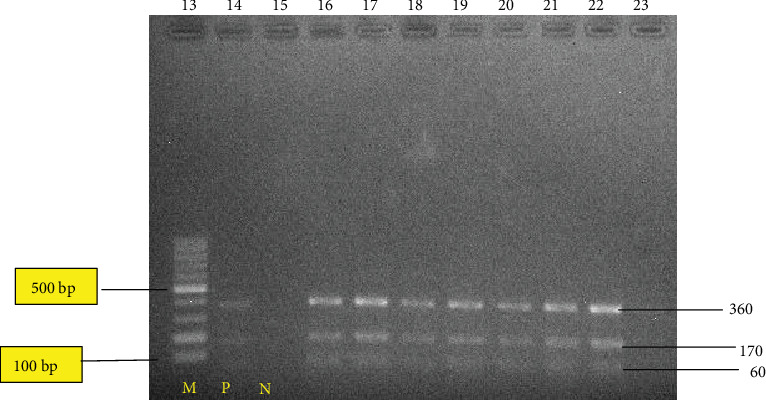
RFLP pattern of PCR product of liver fluke collected from bovine in Shah Alam, Banting, Taiping, and Ipoh abattoirs after digestion with RsaI enzyme. Lane 21: 100 bp ladder molecular; lane 22 positive control; lane 23 is the negative control; lanes 24 to 31: denoted to those of *F. gigantica*. The PCR product digested were run on 2% agarose gel at 100 v for 60 minutes.

**Figure 4:**
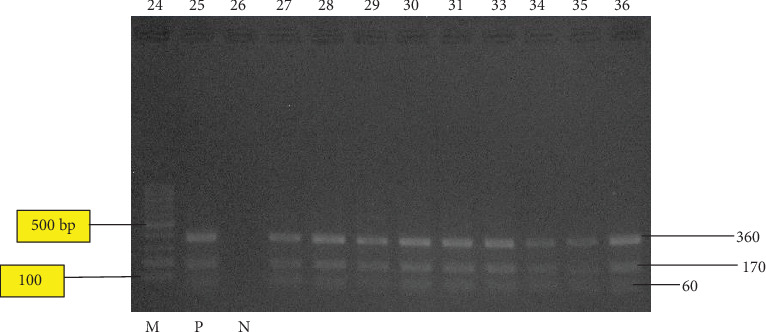
RFLP pattern of PCR product of liver fluke collected from bovine in Shah Alam, Banting, Taiping, and Ipoh abattoirs after digestion with RsaI enzyme. Lane 24: 1000 bp ladder molecular; lane 25 positive control while; lane 26 is the negative control; lanes 27 to 36: denoted to those of *F. gigantica*. The PCR product digested were run on 2% agarose gel at 100 v for 60 minutes.

In this current study, a region of approximately 680 bp of the ITS1 of rDNA in 25 liver samples was successfully amplified (Figures 2–4). However, the negative control did not produce any band on the gels. The results of the PCR product digestion with RsaI were approximately 60, 100, and 360 bp fragments for *F. hepatica*; 60, 170, and 360 bp for *F. gigantica*; and 60, 100, 170, and 370 bp for intermediate form [11]. Based on Figure 2, only *F. gigantica* were reported. Each adult fluke from the 25 condemned liver shows the reading of 60, 170, and 360 bp, which corresponds to *F. gigantica*. These results agreed with the study conducted by Ichikawa and Itagaki [10], who used the RsaI enzyme based on the ITS1 region to distinguish *F. hepatica* and *F. gigantica* in Myanmar. They did not found *F. hepatica* in their studies. There are other researchers from neighbouring countries such as Myanmar and Thailand, who reported the absence of *F. hepatica* [10, 18].

## 4. Conclusion

The occurrence of fascioliasis in Taiping is higher compared to Banting, Shah Alam, and Ipoh abattoirs. The cattle and buffaloes slaughtered at Banting, Shah Alam, Taiping, and Ipoh abattoirs were infected with *F. gigantica* based on the morphometric analysis. Furthermore, a molecular study using the ITS1 marker with the RsaI enzyme proved that *F. gigantica* is the only species that infected cattle and buffaloes in this study area.

## Figures and Tables

**Table 1:** The occurrence of fascioliasis in four slaughterhouses for one year.

Abattoir	Total condemn liver	Total number of the animal slaughtered	Occurrence (%)
Banting	5	2983	0.17
Ipoh	5	392	1.28
Shah Alam	1	3377	0.03
Taiping	14	1014	1.38
Total	25	7,786	2.85

**Table 2:** Comparative morphological data of liver flukes for cattle and buffalo from Banting, Shah Alam, Taiping, and Ipoh abattoirs.

Parameters	Species		*p* value
Measurements (mm)	Cattle *n* = 187	Buffalo *n* = 235	
	Mean ± SD (range)	Mean ± SD (range)	
Body length (BL)	32.31 ± 6.26 (19.30–47.20)	26.41 ± 4.28 (19.20–39.70)	0.000
Body width (BW)	7.69 ± 1.91 (5.00–12.00)	6.77 ± 1.12 (5.00–10.70)	0.000
Cone length (CL)	2.67 ± 0.44 (2.00–4.20)	2.55 ± 0.39 (2.00–3.70)	0.002
Cone width (CW)	2.74 ± 0.49 (2.00–3.90)	2.54 ± 0.40 (1.60–3.90)	0.000
Body area (BA) (mm2) [BL × BW]	253.19 ± 91.13 (108.68–456.50)	179.27 ± 44.51 (105.00–387.84)	0.000
Body perimeter (BP)	75.95 ± 12.11 (53.00–101.00)	64.91 ± 8.34 (50.00–90.00)	0.000
VS-P	29.38 ± 6.07 (17.00–44.70)	23.53 ± 4.04 (16.40–37.40)	0.000
BL/BW ratio	4.37 ± 1.07 (2.50–6.90)	3.99 ± 0.87 (2.52–6.73)	0.000
BL/VS-P ratio	1.10 ± 0.04 (0.84–1.26)	1.12 ± 0.06 (0.84–1.35)	0.000
Body roundness (BR) (mm^2^) [BP]	1.96 ± 0.56 (1.15–3.75)	1.95 ± 0.52 (1.15–3.70)	0.900

**Table 3:** Comparison of morphometric data between *Fasciola hepatica* and *Fasciola gigantica* present in bovines from Egypt [15].

Parameters	Fluke	
	*F. gigantica*	*F. hepatica*
Measurements (mm)	Mean ± SD (range)	Mean ± SD (range)
Body length (BL)	44.65 ± 1.15 (35.25–48.71)	23.73 ± 0.33 (15.48–28.71)
Body width (BW)	10.36 ± 0.46 (8.23–13.60)	10.54 ± 0.15 (8.21–14.27)
Cone length (CL)	3.16 ± 0.11 (2.61–3.68)	2.23 ± 0.04 (1.36–2.98)
Cone width (CW)	3.81 ± 0.10 (3.25–4.34)	3.18 ± 0.04 (2.05–3.99)
Body area (BA) (mm^2^) [BL × BW]	359.20 ± 19.05 (226.16–475.95)	180.92 ± 4.70 (92.73–303.96)
Body parameter (BP)	96.68 ± 2.43 (75.92–104.24)	55.45 ± 0.72 (38.10–69.78)
VS-P	41.02 ± 1.21 (31.01–45.39)	20.79 ± 0.31 (12.40–25.08)
BL/BW ratio	4.37 ± 0.17 (3.43–5.50)	2.27 ± 0.03 (1.65–2.76)
BL/VS-P ratio	1.09 ± 0.01 (1.06–1.14)	1.14 ± 0.004 (1.05–1.28)
Body roundness (BR)	2.10 ± 0.06 (1.76–2.52)	1.37 ± 0.01 (1.15–1.58)

## Data Availability

The datasets supporting the conclusions of this article are included within the article. Raw data are available from the authors upon request.
